# Basal Complex and Basal Venation of Odonata Wings: Structural Diversity and Potential Role in the Wing Deformation

**DOI:** 10.1371/journal.pone.0160610

**Published:** 2016-08-11

**Authors:** H. Rajabi, N. Ghoroubi, M. Malaki, A. Darvizeh, S. N. Gorb

**Affiliations:** 1 Institute of Zoology, Functional Morphology and Biomechanics, Kiel University, Kiel, Germany; 2 Young Researchers Club, Rasht Branch, Islamic Azad University, Rasht, Iran; 3 Department of Mechanical Engineering, The University of Guilan, Rasht, Iran; 4 Department of Mechanical Engineering, Anzali Branch, Islamic Azad University, Bandar Anzali, Iran; CNRS, FRANCE

## Abstract

Dragonflies and damselflies, belonging to the order Odonata, are known to be excellent fliers with versatile flight capabilities. The ability to fly over a wide range of speeds, high manoeuvrability and great agility are a few characteristics of their flight. The architecture of the wings and their structural elements have been found to play a major role in this regard. However, the precise influence of individual wing components on the flight performance of these insects remains unknown. The design of the wing basis (so called basal complex) and the venation of this part are responsible for particular deformability and specific shape of the wing blade. However, the wing bases are rather different in representatives of different odonate groups. This presumably reflects the dimensions of the wings on one hand, and different flight characteristics on the other hand. In this article, we develop the first three-dimensional (3D) finite element (FE) models of the proximal part of the wings of typical representatives of five dragonflies and damselflies families. Using a combination of the basic material properties of insect cuticle, a linear elastic material model and a nonlinear geometric analysis, we simulate the mechanical behaviour of the wing bases. The results reveal that although both the basal venation and the basal complex influence the structural stiffness of the wings, it is only the latter which significantly affects their deformation patterns. The use of numerical simulations enabled us to address the role of various wing components such as the arculus, discoidal cell and triangle on the camber formation in flight. Our study further provides a detailed representation of the stress concentration in the models. The numerical analysis presented in this study is not only of importance for understanding structure-function relationship of insect wings, but also might help to improve the design of the wings for biomimetic micro-air vehicles (MAVs).

## Introduction

Dragonflies and damselflies, belonging to the order Odonata, are among the fastest and most impressive flying insects [1]. They exhibit a combination of unique flight capabilities, which can rarely be observed in other flying insects [2]. Although there is no direct comparison between these two groups among Odonata, one can notice some differences in their flight capabilities. Dragonflies are known as strong fliers, which are capable of long-distance migratory journeys [3]. Some of them, such as *Anax junius*, have been reported to fly up to 150 km per day [4]. In contrast, damselflies are skilful hoverers demonstrating slow, but agile manoeuvrable flight [5].

Recent studies have shown that the aerodynamic lift generation in most insects is strongly influenced by the deformation of their wings during flight [6–8]. The same effect is expected to occur in dragonflies and damselflies [9]. Owing to the pioneering works of Wootton [10], Newman [11], and more recently many other researchers, one can conclude that the deformations exhibited by Odonata wings are mostly passive. This means that the design and the material composition are the main factors that characterize the type and the magnitude of the wing deformation, and consequently the functionality of the whole wing system.

The absence of flight muscles within Odonata wings and their use of passive controls suggest that the wings might serve as potential sources of inspiration in the design of more efficient wings for micro-air vehicles (MAVs). It has already been shown that the fracture toughening mechanisms found in insect wings might lead to the development of more durable and lightweight artificial wings [12, 13]. The material-gradient based design and the presence of the rubber-like material, inspired by wing veins of the dragonfly *Sympetrum vulgatum*, were suggested to improve the damping properties of dynamic systems [14]. The use of the same mechanisms controlling the deformability of Odonata wings might improve the flight performance of artificial flapping-wing vehicles. What are the mechanisms contributing to the passive control of wing deformations?

**Fig 1:**
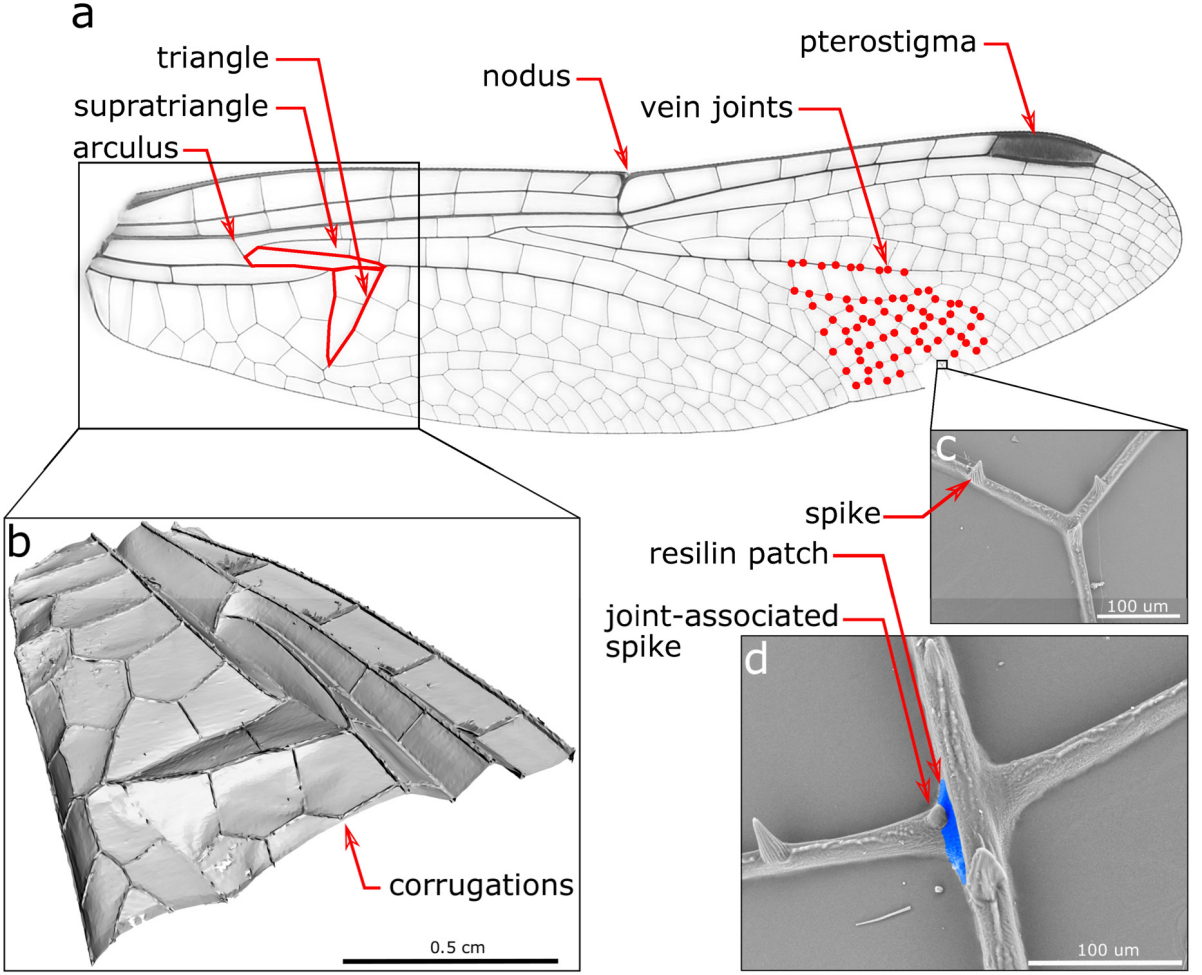
(a) Main structural components of the forewing of the dragonfly *S*. *vulgatum* (Libellulid); (b) 3D geometry of the basal complex; (c, d) scanning electron microscopy images of spikes, a joint-associated spikes, and a resilin patch.

To the best of our knowledge, the characteristics influencing the deformability of Odonata wings are known to be: (i) inhomogeneous material distribution [15, 16], (ii) non-uniform thickness [17, 18], (iii) venation pattern [19–21], (iv) corrugation [22, 23], (v) nodus [20, 24], (vi) pterostigma [25], (vii) vein joints [11, 26–29], (viii) joint-associated spikes [28, 29], (ix) resilin patches [27, 30, 31], (x) vein ultrastructure [14, 32], (xi) vein fractures [10], (xii) flexion lines [10, 33], and (xiii) basal complex [34, 35]. Fig 1 illustrates the main structural elements of the forewing of the dragonfly *S*. *vulgatum*. What is the quantitative influence of each individual element on the deformation of Odonata wings?

Although now we know much more about the role of structural elements in the biomechanics of Odonata wings, the precise effect of the individual elements is still not well understood. Compared to all other wing components, the complex structure of the proximal part of the wing, known as the “basal complex”, has been less studied until now. This three-dimensional (3D) corrugated structure at the base of the wing is a functional unit which generally consists of a set of relatively thick veins, arculus, discoidal cell, triangle and supratriangle (the lower and upper parts of the discoidal cell, respectively).

In 1989, Wootton and Ennos described the function of the arculus in the wings of Diptera [36, 37]. Using a simple card model, they reproduced the deformation of the wings in flight. The results of these studies suggested the strong contribution of the arculus to the wing twisting. Later, Wootton extended this research to the wings of Odonata [20]. The flight skills derived from morphological differences between the wings of dragonflies and damselflies were explained by him in detail. His article is the first comprehensive description of the effect of the basal complex on the odonate wing deformation. The use of the card models was further continued by him and his co-workers and was demonstrated to be a powerful methodology for this kind of questions [34, 35]. The models were utilized to simulate the wing deformation in various odonate species, which helped to identify and interpret the mechanisms involved.

These observations encouraged us to design the present computational study, in order to obtain comparative quantitative measurements of the deformation of the wing basal complex. Considering the limitations of the card models, such as the difficulty in forming, including the veins and assigning appropriate material properties, this study was undertaken to provide deeper insights into the deformation pattern and the stress distribution within the proximal part of Odonata wings. Using a series of finite element (FE) models, we further extend our previous studies [13, 14, 21, 28, 29] on the investigation of the effects of different wing components on the wing deformation. The term “basal venation” is used here to refer to the venation pattern of the basal region.

## Materials and Methods

### Finite element modelling

**Fig 2:**
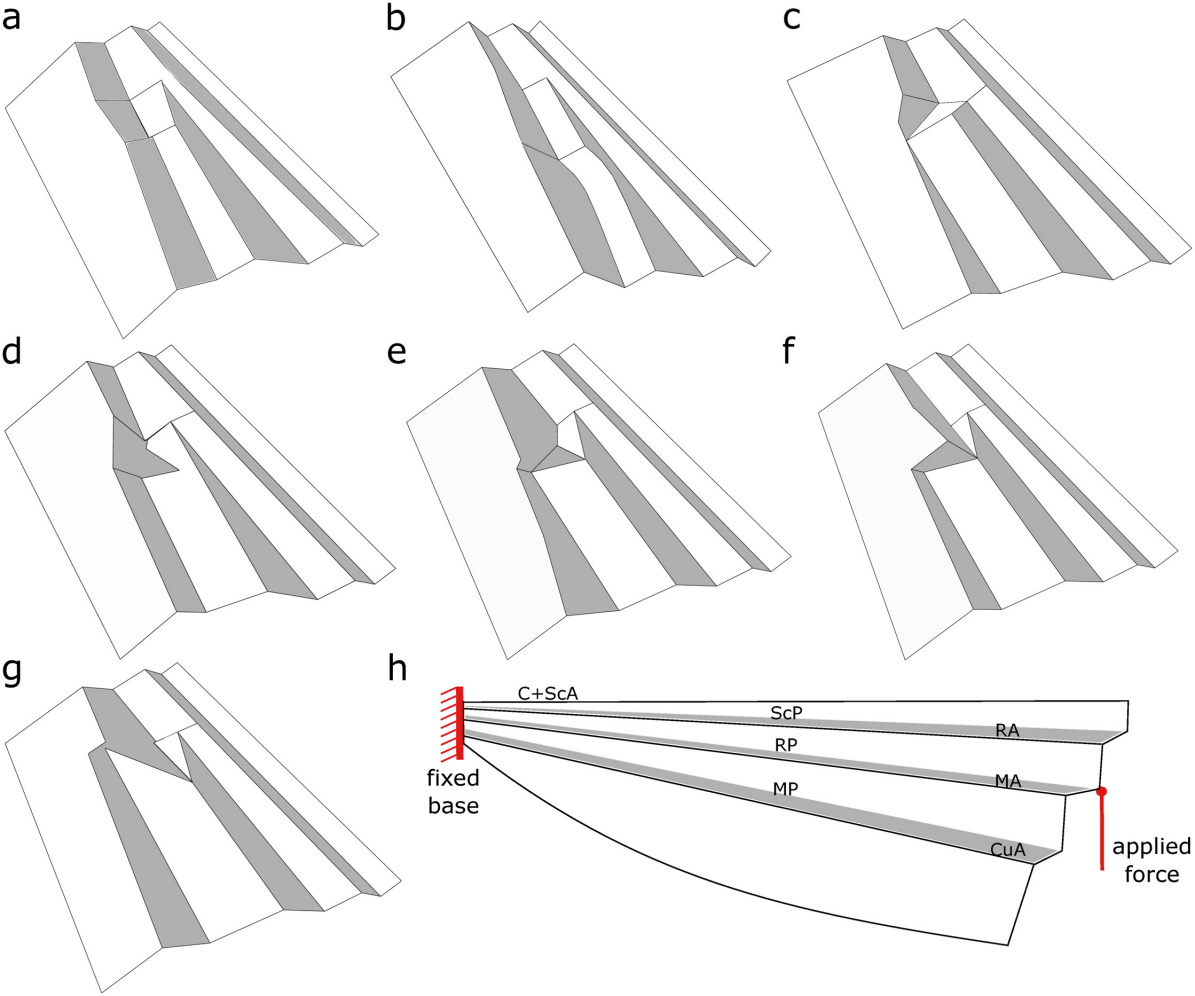
3D configuration of the basal complex in the FE models of wings. The models were inspired by (a) Coenagrionid wings, (b) Chlorocyphid wings, (c) Heterophlebia forewing, (d) Heterophlebia hindwing, (e) Aeshnid forewing, (f) Libellulid forewing, (g) Libellulid hindwing (see Wootton and Newman, 2008). (h) Schematic view of a model under ventral loading. The external force is applied to RP, and the model is fixed at the wing base.

The commercial FE software package, ABAQUS/Standard version 6.14, was used to develop 3D numerical models of the proximal part of Odonata wings. The developed models can be categorized in three main groups:

1. Group 1 includes the most comprehensive models, representing several structural features of a real wing. The models of this group contain both the 3D configuration (basal complex) and the venous structure (basal venation). Considering that most of the vein joints in the basal part are rigid containing no resilin patch, all crossing veins in our models were assumed to have rigid contacts. Corrugation angles in each model were considered to be similar to those of the card models presented by Wootton and Newman [34], which are estimates of those of real insect wings. As an example, the model shown in Fig 2f can serve as a simplified model of the forewing basal complex of the dragonfly *S*. *vulgatum* (Fig 1), which belongs to the family Libellulidae. However, it should be mentioned that the 3D configuration of the basal region may slightly vary from one species to another in an individual family. Therefore one may observe small differences between the developed models and the wings of individual species from the same family. The models of Group 1 were used as reference models in our study.
2. The models of Group 2 were developed by excluding the veins from the models of Group 1. In other words, the models in Group 2 have a corrugated structure, but no veins.
3. The removal of the 3D configuration from the models of Group 1 resulted in the models of Group 3. Hence, the models in the latter group are flat surfaces, containing the detailed venation pattern of real wings.

Each of the three above mentioned groups include seven wing models. These models were previously selected from the typical representatives of the five Odonata families, reviewed and discussed by Wootton and Newman [34]. The models are as follows: (i) Coenagrionid wings, (ii) Chlorocyphid wings, (iii) Heterophlebia forewing, (iv) Heterophlebia hindwing, (v) Aeshnid forewing, (vi) Libellulid forewing, (vii) Libellulid hindwing. Considering the relatively similar 3D configuration of the basal complex of fore- and hindwings in Coenagrionid (Zygoptera), Chlorocyphid (Zygoptera) and Aeshnid (Anisoptera), we modelled only forewings of these families. Fig 2a–2g shows the 3D structure of the basal complex in the models of Group 1. The developed models have the same length of 1 cm, but their widths vary depending on the geometry of the corresponding real wings. The input files of the created models of Group 1 can be found in S1–S7 Models.

In all the developed models the thickness of the membrane was taken to be constant and equal to 2 μm over the entire wing proximal part [17, 38]. The wing veins were modelled as having a circular cross-section. The radius and the wall thickness of veins were assumed to linearly decrease from the wing base to the free end of the models. The maximum and minimum vein radii were chosen to be 35 μm and 25 μm, respectively [23]. The vein thickness was also considered to have maximum and minimum values of 23 μm and 17 μm, respectively [23].

The general-purpose linear four-node shell elements with reduced integration (S4R), which are suitable for a wide range of applications, were employed for modelling of the wing membranes [39]. The use of the reduced-integration elements results in a significantly reduced computational time. The wing veins were modelled using shear-deformable three-node beam elements (B32). These elements, regarding their second-order formulation, provide higher accuracy in comparison with other beam elements. An enhanced hourglass control was further utilized to avoid uncontrolled distortions arising from the presence of first-order shell elements.

In order to eliminate the effect of the element size on the results, a mesh convergence analysis was conducted for individual models under each loading condition. An element size of 0.015±0.009 mm was obtained by performing the analysis for a reasonable mesh density and then decreasing the size of the elements and repeating the analysis. This process continued until obtaining the results which were independent from the mesh size.

### Material properties

Our previous numerical simulations showed that the use of a linear elastic material model in a simple, but accurate, FE model enables us to simulate the complex mechanical behaviour of insect cuticle [13, 14, 21, 28, 29]. In this study, we used the basic material properties of insect cuticle. The cuticle of the wing was assumed to be isotropic, with a Young’s modulus of 3 GPa [40]. The wing strength was chosen to be 27.79 MPa, which corresponds to the tensile strength of the wings of the desert locust *Schistocerca gregaria* [12, 41]. A density and a Poisson’s ratio of 1200 kg/m^3^ and 0.49 were assigned to both veins and membranes, respectively [42, 43].

It is also important to note that we used the same material properties for all the developed models. This modelling strategy allowed us to eliminate the effect of possible differences in the properties of the wings on their mechanical behaviour, even if such differences might be well possible in real wings. In other words, the only variables in the models presented here are the 3D structure and structural components.

### Loading and boundary conditions

This article presents two comparative studies between the mechanical behaviours of (i) the corresponding models of the three different wing groups (Groups 1–3) and (ii) the different wing models within Group 1 (realistic models having both the venation pattern and the pleated structure). Although the same loading conditions were used in both analyses, we had to utilize different loading magnitudes in each one. The reason can be found in the reduced stiffness of the models in both Groups 2 and 3.

Wootton et al. [35] have already shown that the aerodynamic forces in flight are mainly concentrated in a region of the wing adjacent to the posterior radial vein (RP). Therefore, applying a point force to RP can result in the natural deformation of insect wings in flight [11, 20]. Here, we employed the same method to reproduce the lift generated by the wings during strokes.

Taking into account that our models represent a small region from the proximal part of insect wings, we utilized a mechanical load of 0.19 mN. This force is about one-third of that exerted by the insects’ weight on each wing (measured for the dragonfly *Sympetrum frequens*) [44]. The use of the mentioned force on both dorsal and ventral sides of RP enabled us to simulate the deformation of the wings in both up and down strokes.

The preliminary results indicated that the application of the same force to the models of Groups 2 and 3 may lead to extremely large deformations. Therefore, in order to make a comparison between deformations exhibited by the corresponding models from the three different groups, we had to decrease the magnitude of the applied force. For this comparative study, a point force of 0.06 mN was employed.

In all simulations, the wing models were assumed to be fixed at the base (clamped boundary condition). Considering the ability of insect wings to resist moments, it seems likely that the selected boundary condition represents the typical constrains at the wing base [45]. Fig 2h shows a schematic view of a wing model together with the approximate position of the applied force and the indicated boundary condition.

In this study, the implicit solver ABAQUS/Standard was utilized for the computational analysis. The use of the implicit scheme leads to highly accurate deformation/stress solutions [39]. Taking into account that Odonata wings may experience large deformations in flight, we further employed a nonlinear geometric analysis. Consideration of the geometric nonlinearity allowed us to include the effect of the changes in the wing geometric configuration during deformation.

Although the application of a nonlinear analysis lead to higher accuracy of the results, due to the excessive computational costs, the use of this analysis was limited to the comparative study between the individual models in Group 1. On the other hand, we have found that the effects of the removal of the basal complex and the basal venation on the mechanical behaviour of the models were much higher than the effect of the analysis type. Therefore, for the comparative study between the different groups, a linear geometric analysis was utilized. It is believed that the type of analysis (linear/nonlinear) does not alter the conclusions reached in the latter comparative study.

## Results

**Fig 3:**
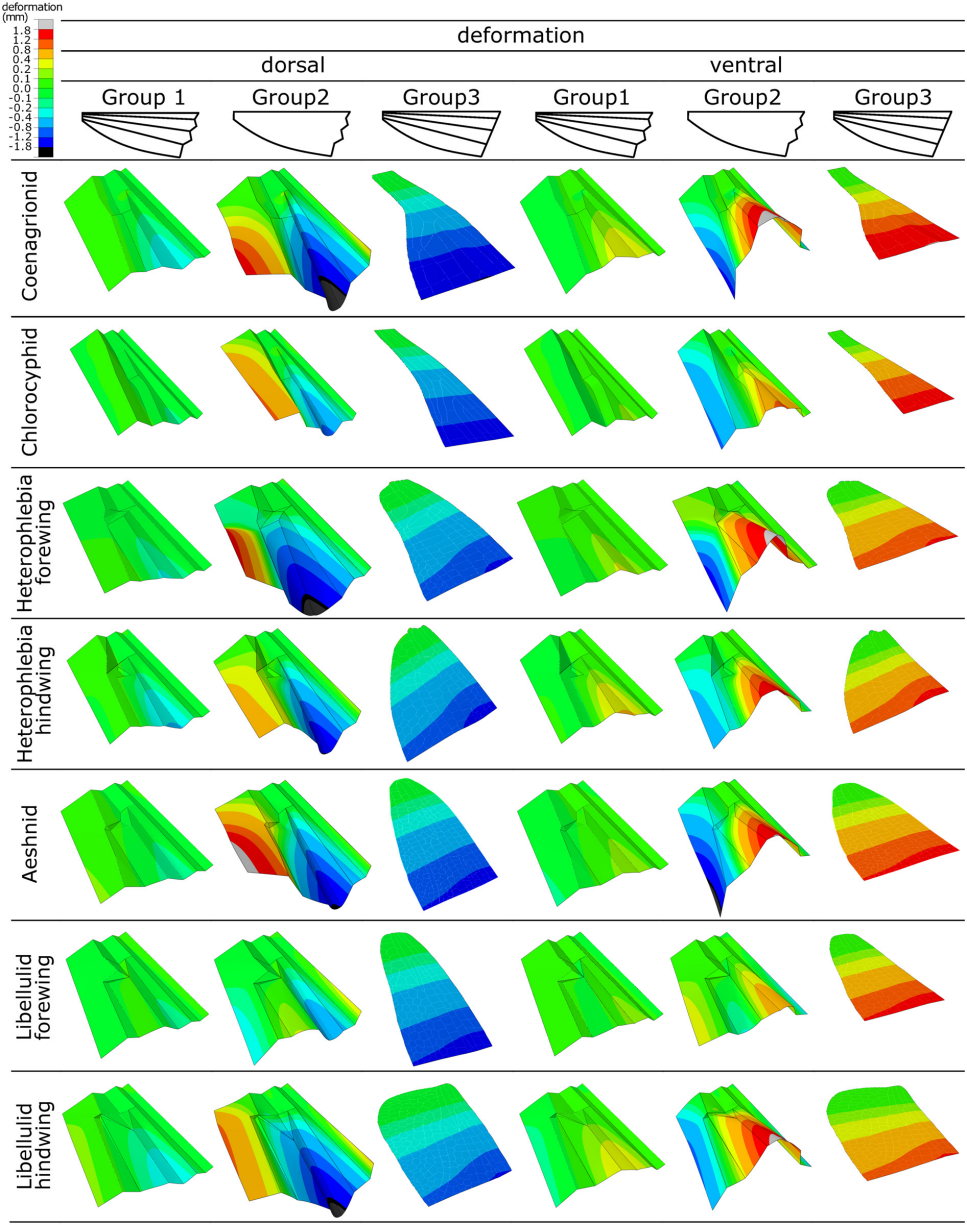
Deformation pattern of the models of the three model groups. The wing deformation has been shown under both dorsal and ventral loadings. The removal of the basal veins from the models of Group 1 led to the significantly larger deformations in the models of Group 2. However, the corresponding models from these two groups show a relatively similar deformation pattern. The models from Group 3 (models without basal complex, but containing basal veins) experience much larger deformation than the models of Group 1. The totally different types of the deformation in the corresponding models from these groups indicate the crucial influence of the basal complex on the deformation pattern of Odonata wings.

Fig 3 represents the deformation pattern of the models, belonging to the three developed wing groups, under both dorsal and ventral loadings. As seen in this figure, models from Group 1 (realistic models), in comparison to the models from the other two groups, experience considerably small displacements under the same applied force. The maximum bending displacement of the models from Group 2 (models without basal venation) is at least 4.3 times and at most 9.6 times larger than that of their corresponding models in Group 1 (realistic models). In some cases, the difference in the magnitude of the maximum displacement is even more pronounced between the corresponding models of Groups 1 (realistic models) and 3 (without basal complex). The models of Group 3 are seen to have maximum displacements 3.4–11.4 times larger than those of the models of Group 1.

Although the models of Group 2 (models without basal venation) undergo considerably larger deformations than those from Group 1 (realistic models), a relatively similar deformation pattern is observed in the corresponding models from the two groups. As seen in Fig 3, this deformation is a combination of bending and torsion that mainly occurs in the region around RP and at the posterior part of the models, respectively. In the models from both groups, the leading edge either is not deformed or only undergoes very small deflection.

In contrast, the models in Group 3 (models without basal complex) exhibit a very different type of deformation. The deformation of these models is dominated by bending, in which all points located along a given cross-section have almost the same displacement (see Fig 3). In this deformation regime, a uniform bending, but no twisting, occurs along the length of the models and the maximum displacement takes place at their free ends.

**Fig 4:**
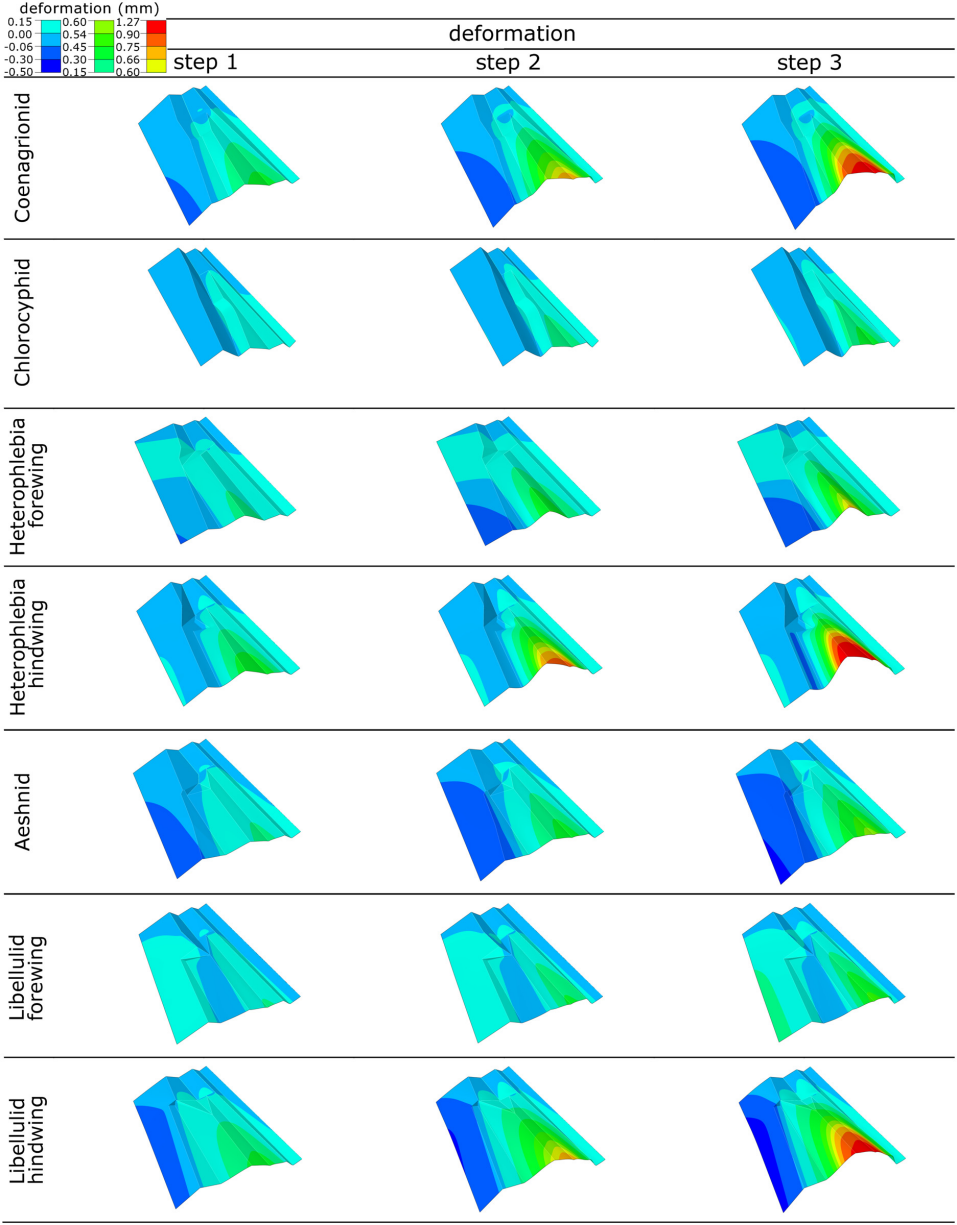
Selected snapshots of the deformation pattern of the realistic wing models (Group 1). The different configurations of the basal complex result in the different types and magnitudes of the deformation.

Fig 4 shows three representative snapshots of the deformation process in the realistic models (Group 1). The deformation pattern of the models shows that applying a mechanical load to the ventral side of the wing, simulating the aerodynamic lift, pushes RP up. The rise of RP leads to the upward movement of its adjacent longitudinal veins, the anterior radial vein (RA) and the anterior medial vein (MA). This effect results in the bending deformation of a surface of the wing that the mentioned veins lie on. The rise of MA affects the deformation of the posterior part of the wing, so that when it rises, the discoidal cell undergoes a counterclockwise rotation. In all wing models, except in the Chlorocyphid one, the hindwing of Heterophlebia and the Libellulid forewing, this effect leads to the downward displacement of the wing trailing edge. In the three exceptions, the trailing edge slightly arises and moves laterally as the discoidal cell rotates. Although the trailing edge in these three models shows no downward displacement, they still have a tendency towards camber formation. Comparison of the deformation patterns of the models suggests that the Libellulid hindwing and the Chlorocyphid wings exhibit the highest and lowest abilities to develop a cambered section, respectively. The gradual deformation of the models of Group 1, under ventral loading, is shown in S1–S7 Videos.

Although the deformation pattern of the wing models in the dorsal loading is totally different from that we have observed in the ventral loading (see Fig 3), a basically similar deformation mechanism operates in both loading conditions. A force applied to RP from above depresses this vein and its adjacent longitudinal veins (RA and MA). This effect consequently causes the region between RA and MA to deflect downwards. The clockwise rotation of the discoidal cell, due to the lowering of MA, lifts up the free end of the trailing edge. Similar to the ventral loading, the trailing edge in the Chlorocyphid wings, the Heterophlebia hindwing and the Libellulid forewing undergoes a deformation in the lateral direction. The leading edge in all the developed models from this group shows only a very small displacement under both dorsal and ventral loadings.

**Fig 5:**
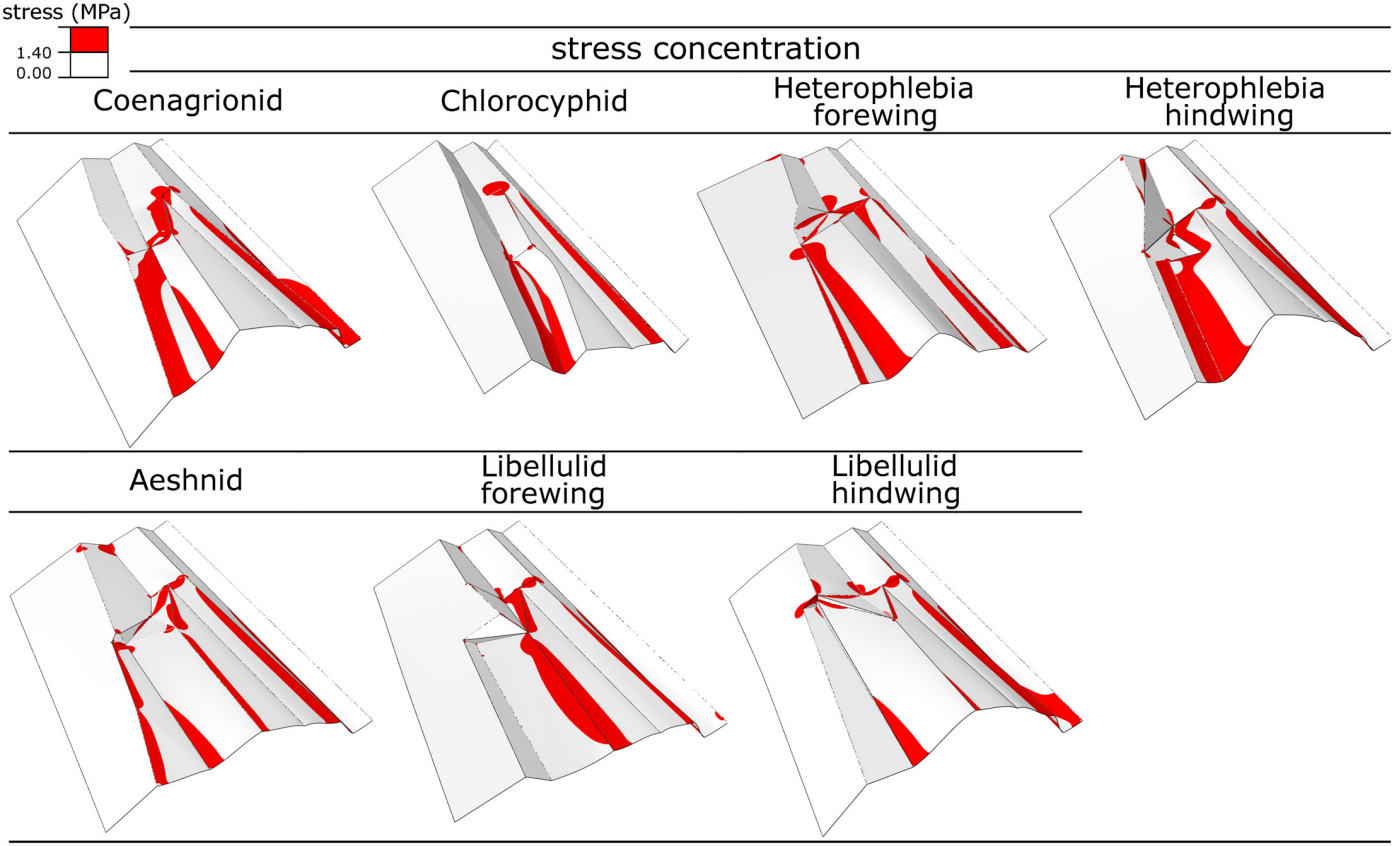
Localized stress in the realistic models (Group 1). The models are subjected to 0.19 mN point force applied to RP from the ventral side. The regions with the maximum principal stress larger than 5% of the ultimate tensile strength of the wing material are marked with the red colour.

The concentration of the maximum principal stress in the realistic models (Group 1) subjected to the ventral loading (0.19 mN force applied to RP) is presented in Fig 5. The red colour shows the regions with stresses larger than 5% of the strength of the wing material. When the mechanical load is applied to the models, the first stress concentration occurs around the arculus. The increase in the magnitude of the force leads to an increase in the local stress in the wings and distributes the high mechanical stress over a larger area. In all models, in addition to the arculus, the maximum stress can be seen near the discoidal cell and in the regions adjacent to some of the longitudinal veins.

## Discussion

### The effect of the basal complex

It has been already stated that the wing corrugations result in an improved dynamic stability of insect flight. This effect is caused not only by increasing the lift-to-drag ratio at all Reynolds numbers [22, 46, 47], but also by increasing the ratio of the resonance to the flapping frequency [21, 48, 49]. The latter appeared to be due to the role of the corrugations in enhancing the structural stiffness of the wing. The same effect has been observed here in the presence of the 3D configuration of the proximal part (see Fig 3). Hence, considering this increased structural rigidity one might expect that the basal complex can further avoid extremely large deformations of Odonata wings under a given force.

Considering the relatively large deformation of the leading edge in the models from Group 3 (models without basal complex), it can be concluded that the basal complex may further play a role in the enhanced rigidity of the anterior part of the wing. The presence of a stiff anterior margin, as the main part of the supporting area in many insect wings, improves their load-bearing capacity [21] and prevents them from fluttering [35, 50]. We would also like to mention that, leading edge in some Odonata may undergo much larger deformation than that predicted by our computational models. For example, we have observed very large displacements in the leading edge of the small damselfly *Agriocnemis femina* during the take-off phase of flight (unpublished data). The same phenomenon has been reported by Wootton [10] and Ennos [37] in the wings of many Diptera. However, one should consider that the large deformations typically occur in the distal part of the leading edge and not in its proximal part studied here.

In addition to the increased rigidity, the basal complex may contribute to the deformation mechanism of Odonata wings. The computational results showed that the removal of the basal complex from the wing models causes a transition in the type of deformation from a combination of bending and twisting in Group 1, to pure bending in Group 3 (see Fig 3). Hence, it can be expected that the wing with a corrugated base has a reduced torsional stiffness. As illustrated by the realistic models of Group 1, the twisting of the wing helps to form a cambered section in flight [19]. The latter is of great importance, since it may have a strong influence on the flight performance of the insect [51]. A cambered aerofoil, such as that found in most insect wings, allows for high lift production beyond the capacity of a flat plate [52]. Taking into account that insect wings have to withstand high bending moments in flight, a cambered wing can further provide an additional bending stiffness [10].

Experimental results revealed a large asymmetry in the flexural stiffness of insect wings subjected to forces acting on the dorsal and ventral sides [19, 42, 53–55]. The vein joints [21, 28], nodal lines [10], and wing camber [54] were supposed to be potential sources of this asymmetry. In our simulations, we have observed a strong anisotropy in the deformation of the models under dorsal and ventral loadings. Such an asymmetry may come from the presence of the basal complex, which results in the change of the wing cross-section depending on the direction of the bending. Therefore, the second moment of area of the wing section would not be constant, but would be a function of the wing deformation. In other words, the different wing cross-sections under dorsal and ventral loadings cause the difference in the second moment of area, as a measure of the resistance to bending. Combes and Daniel stated that they failed to reproduce this dorsal/ventral asymmetry by adding a cambered section in their numerical models [42]. The reason can be found in the use of a linear geometric analysis, which results in a constant second moment of area.

Interestingly, the bending deformation in the realistic wing models (Group 1) occurs about the arculus, and not about the wing base. Indeed, the base of the wing, in all the developed models of this group, undergoes almost no deformation. Considering the stress distribution within the realistic wing models (see Fig 5), it is highly likely that the stiff part around the arculus acts as a mechanism which further avoids the transfer of the stress to the wing base. This effect may prevent the extra energy consumption in flight muscles to resist the additional mechanical stresses transmitted by the wing. It is also important to note that, in real wings, the high stress around some longitudinal veins may be remarkably reduced by the presence of compliant resilin patches [26, 28, 29].

### The effect of the basal venation

Comparison of the deformation of the models from Group 1 (realistic models) and Group 2 (models without basal venation) indicates that the veins from the basal part of the wing remarkably enhance its structural stiffness. However, considering the relatively similar deformation pattern of the corresponding models in these two groups (see Fig 3), it can be concluded that the basal veins do not effectively influence the mechanism of the wing deformation. The obtained numerical results are in good agreement with our previous findings, indicating the large increase in the deflection of the wings of the dragonfly *Orthetrum sabina* due to the removal of the veins, which is associated without significant change in their deformation pattern [21].

Although, in order to generate an optimum lift force, the whole wing structure should be flexible enough [56], the rigidity of the wing base, which makes the supporting region of the insect wing, has particular importance. The basal part of the wing is usually subjected to large bending and torsional moments, compared to the other wing areas. Hence, the additional rigidity of this region obtained by the presence of the relatively thick basal veins gives the wing the ability to withstand aerodynamic forces without buckling. The authors have previously reported that veins in an insect wing may also provide benefits to the insect by distributing the stress and preventing stress concentrations [13, 21, 57]. The veins further effectively resist the propagation of an induced crack by increasing the structural fracture toughness of the whole wing structure [12, 13].

### Comparative wing deformation in Odonata

Although there are quantitative differences in the deflection of the wings of the five Odonata families investigated in this study, they utilize either one or both of the following mechanisms of deformation: (i) cambering and (ii) widening.

The wing camber can be defined as a relatively large bending in the region around RP, RA and MA, which pushes down the trailing edge and leads to the wing twisting. Therefore, the wing camber is not only the result of the twisting ability of the wing (as already known), but is the function of both the wing twisting and bending.

In contrast, the wing widening is characterized by the opening of the corrugations and the flattening of the wing, which results in the lateral displacement of the trailing edge. Therefore, in the widening regime, the wing structure seems to be laterally stretched rather than bent or twisted.

Both the cambering and widening may help the insect to increase the wing effective surface area, which consequently improves the aerodynamic force generation. However, one can expect that the camber formation, in addition to the higher flexural stiffness, results in more efficient lift production, compared to the wing widening. Such a “high-lift” design seems to be more important for those Odonata classified as perchers, exhibiting a slow and an “up and down” flight. Among all the dragonfly families investigated in this study, libellulids are the only species which appeared to be perchers [58]. Interestingly, the Libellulid hindwing, similar to the wings of Coenagrionid, was found to generate a highly cambered section (see Fig 4). However, the wing models belonging to flier dragonflies become only slightly cambered according to ventral loading. It is noteworthy to mention that the higher lift generated by a highly cambered wing may lead to a lower wing-beat frequency and consequently a lower energy consumption, which are the flight characteristics of perchers.

The magnitude of the wing deformation and the functioning of both deformation mechanisms remarkably vary among the studied Odonata families (see Fig 4). As already mentioned, Coenagrionid wings and the Libellulid hindwing are good examples of camber formation. In contrast, the deformation of the other models is a combination of mainly widening and, to a lesser degree, cambering. We have also observed considerable differences between the deformations of the models of fore- and hindwings of an individual family. Therefore, considering the more significant role of the basal complex on the deformation mechanism of the models compared to the basal venation, one can assume that the different configurations of the basal complex in these insects may influence the overall deformability of their wings in flight. The latter may consequently result in apparent differences in the flight styles of these Odonata families.

The comparison of the deformations of the developed wing models suggests that the use of the following design strategies may facilitate the generation of a cambered section in flight: (i) the presence of a stiff arculus, (ii) a large and posterior-distally inclined discoidal cell, (iii) a large triangle extended towards the wing trailing edge, (iv) an obtuse angle between the plates located on the right and left sides of the posterior medial vein (MP), and (v) posterior-distally oriented medial veins (MA and MP). All these structural features, except the first one which acts as a support for the bending of the middle wing area, allow the force transmission to the posterior wing area and pushing the trailing edge down.

The narrow and highly corrugated proximal part, in which the longitudinal veins are located very close to each other, seem to be the main reason of the reduced ability of the Chlorocyphid wings to depress the trailing edge. However, it can be seen that, in the real wing, this weak tendency towards twisting of the trailing edge has been counterbalanced by the position of the nodus along the leading edge. Indeed, in Chlorocyphid wings, similar to those of many other damselflies, the nodus lies more closely to the wing base than to the wing tip. It is expected that the position of the nodus along the anterior margin, in the wings of damselflies, is highly correlated with the wing twisting in flight [20].

The deformation of insect wings is, without doubt, the result of the complex mechanical interactions between different wing components. Consideration of the role of different wing elements in isolation may under- or overestimate their influence on the mechanical behaviour of the wing. However, in order to understand the effect of each element, a systematic study on individual wing components, such as that presented here, appears to be an efficient approach. Following our previous studies [21, 28, 29], this article presents a further step towards the realistic modelling of insect wings. The comparative analysis carried out in this article provides better insights into the functional morphology of the proximal part of Odonata wings. However, the detailed influence of many other wing features is still not very well understood. Therefore, our future works will focus on the numerical modelling of such structural elements as the nodus and pterostigma.

## Conclusion

This article presents the first numerical investigation of the effect of the basal complex and basal venation on the mechanical behaviour of Odonata wings. Using a series of FE models, inspired by the previous studies on the wings of five Odonata families, we show that both the mentioned types of structural characteristics result in higher flexural stiffness of the wing. However, it is only the basal complex which dominates the particular deformation pattern of the wings in flight. The basal complex is further expected to contribute to the dorsal/ventral asymmetry in the wing deformation. The comparative analysis of the deformation behaviour of the wing models of different odonate groups enabled us to investigate the effect of the 3D configuration of the basal complex on the wing camber formation. Our numerical models were also found to be capable of predicting the local stress concentrations in the wings. The findings of this study can be potentially used to improve the performance of bio-inspired wings for MAVs.

## Supporting Information

S1 Video Deformation process of the proximal region of the Coenagrionid wings under ventral loading. (ZIP)

S2 Video Deformation process of the proximal region of the Chlorocyphid wings under ventral loading. (ZIP)

S3 Video Deformation process of the proximal region of the Heterophlebia forewing under ventral loading. (ZIP)

S4 Video Deformation process of the proximal region of the Heterophlebia hindwing under ventral loading. (ZIP)

S5 Video Deformation process of the proximal region of the Aeshnid wings under ventral loading. (ZIP)

S6 Video Deformation process of the proximal region of the Libellulid forewing under ventral loading. (ZIP)

S7 Video Deformation process of the proximal region of the Libellulid hindwing under ventral loading. (ZIP)

S1 Model FE model of the basal complex of Coenagrionid wings. (STL)

S2 Model FE model of the basal complex of Chlorocyphid wings. (STL)

S3 Model FE model of the basal complex of the Heterophlebia forewing. (STL)

S4 Model FE model of the basal complex of the Heterophlebia hindwing. (STL)

S5 Model FE model of the basal complex of Aeshnid wings. (STL)

S6 Model FE model of the basal complex of the Libellulid forewing. (STL)

S7 Model FE model of the basal complex of the Libellulid hindwing. (STL)
